# Influence of Social Media on Medication-Related Decision-Making Among Adults in Saudi Arabia: A Cross-Sectional Study

**DOI:** 10.7759/cureus.111272

**Published:** 2026-06-22

**Authors:** Emtenan M Alharbi

**Affiliations:** 1 Department of Clinical Pharmacy, College of Pharmacy, King Saud University, Riyadh, SAU

**Keywords:** health information, medication choices, online health behavior, saudi arabia, social media

## Abstract

Background: Social media has become an integral part of modern communication, potentially influencing health-related decisions, including medication choices. This study aimed to investigate the role of social media in medication choices among adult users in Saudi Arabia.

Methods: A cross-sectional descriptive study was conducted via an online, self-administered Arabic questionnaire to recruit adult social media users in Saudi Arabia through convenience sampling.

Results: A total of 742 participants completed the survey, including 541 (72.9%) female and 201 (27.1%) male participants. The questionnaire demonstrated good reliability (Cronbach's alpha = 0.87). Significant positive correlations were found between time spent on social media and the frequency of searching for health-related information (rho = 0.18, p < 0.001) and medication-related information (rho = 0.19, p < 0.001). While 430 participants (58.0%) preferred consulting physicians for medication information, 299 participants (40.3%) reported that medication-related social media posts sometimes or frequently influenced their medication-use decisions. Among female participants, age was positively correlated with the perceived reliability of advertisements promoting medicines on social media (rho = 0.122, p = 0.005), and education level was correlated with concern about the accuracy of information found on social media about medications (rho = 0.094, p = 0.028).

Conclusion: Social media use was associated with medication-related decision-making among adults in Saudi Arabia, with notable gender differences. These findings highlight the need for healthcare professionals and policymakers to develop effective strategies to address the role of social media in medication-related decisions.

## Introduction

Social media has become an integral part of daily life for millions worldwide, including in Saudi Arabia. With approximately 76.8% of the Saudi population using at least one social media platform and spending an average of three hours and 10 minutes daily on these platforms, the influence of social media extends to health behaviors and decisions, including medication choices [1].

Social media platforms provide unprecedented access to health information, enabling users to learn about medications, share experiences, and seek advice outside traditional healthcare settings [2]. While this accessibility can empower individuals with knowledge, it also raises concerns about the accuracy and reliability of such information.

Previous research has highlighted the complex relationship between social media and medication choices. Studies have shown that social media can provide valuable information and support for individuals with health issues but can also expose users to misinformation and potentially influence medication decisions without proper medical guidance [3,4]. A previous study reported that many YouTube videos on prescription medications presented positive portrayals with limited mention of adverse effects [5].

In Saudi Arabia, previous studies have investigated related issues such as media exposure and substance use among young adults and social media addiction and self-medication [6,7]. However, the wider Saudi literature on social media and medication-related information continues to evolve, and further evidence is needed to clarify how online exposure relates to practical medication decisions among adult users. In addition, gender may influence how individuals seek, evaluate, and respond to online health information [8]. The present study, therefore, focuses on medication-related decision-making, including information-source preference, trust and accuracy concerns, self-reported behavioral changes, and gender-specific patterns.

This study aims to address this gap by investigating the role of social media in medication choices among adult users in Saudi Arabia, with attention to demographic factors, including gender differences.

## Materials and methods

Study design and participants

A cross-sectional descriptive study was conducted from January to March 2024 among adult social media users in Saudi Arabia.

Participants

Participants were recruited using convenience sampling through a survey link posted across various social media groups. Eligible participants were adult social media users aged 18 years and above, of either gender, willing to participate in the survey, and able to read and understand Arabic. Participants who did not reside in Saudi Arabia or were unable to read or understand Arabic were excluded.

Sample size calculation

The minimum required sample size was estimated using Cochran’s formula for cross-sectional studies, assuming a 95% confidence level, 5% margin of error, and a conservative expected response distribution of 50% because no precise prior estimate was available for the main outcome. The minimum required sample size was 385 participants. To improve precision and account for incomplete responses, the main survey remained open until 742 complete eligible responses were obtained for the final analysis. The final analytical sample, therefore, included 742 complete responses, excluding the 50 pilot responses.

Survey instrument

An online, self-administered Arabic questionnaire was developed and distributed using Google Forms (Google LLC, Mountain View, CA) through social media platforms, including Twitter (X Corp., San Francisco, CA), Facebook (Meta Platforms, Menlo Park, CA), and WhatsApp (Meta Platforms). The questionnaire was developed following essential steps for survey development and validation as described by Krosnick and Presser [9] and Weber et al. [10]. The Arabic questionnaire was the version administered to participants (see Appendix A); an English translation is provided in Appendix B for reader reference. The survey included demographic characteristics, social media usage patterns, and medication-related choices and behaviors. Demographic items included gender, age, marital status, education level, residency region, current job, monthly income, and presence of chronic diseases. Social media usage items assessed preferred platforms, daily time spent on social media, frequency of searching for health- and medication-related information, sharing medication-related information, and consulting health communities online. Medication-related items assessed regularly used medications, reliance on social media for drug-purchasing decisions, the influence of medication-related posts on personal decisions, trust in social media information, behavioral changes based on social media information, and attitudes toward medication information on social media.

Responses were recorded using a 5-point Likert scale for most items. The questionnaire was reviewed by five experts with backgrounds in clinical pharmacy, epidemiology or public health research, and statistics. The experts assessed the questionnaire for clarity, relevance, face validity, content validity, wording, response options, and alignment with the study objectives. Minor wording and formatting modifications were made before distribution to improve clarity and readability.

Because the questionnaire included mixed item types and was intended to describe behaviors and attitudes rather than measure a single latent construct, factor analysis was not performed.

Pilot study and reliability

A pilot study was conducted with 50 respondents before the main survey distribution to assess questionnaire clarity, completion time, and internal consistency. Feedback from the pilot phase was used to make minor refinements to wording and formatting. Pilot responses were excluded from the final analysis. The questionnaire’s overall reliability was assessed using Cronbach’s alpha, with a score of 0.87 indicating good internal consistency. Domain-based reliability coefficients were not calculated separately because the questionnaire included mixed item types and was designed to describe medication-related behaviors, attitudes, trust, influence, and information-seeking patterns rather than to function as a validated multidomain psychometric scale. The pilot survey was distributed using the same online questionnaire format and through the same planned social media platforms as the main survey.

Ethical considerations

Ethical approval was obtained from the Standing Committee for Scientific Research Ethics, King Saud University (approval no. KSU-HE-23-1083). Before starting the online questionnaire, participants were shown an information page explaining the purpose of the study, the voluntary nature of participation, confidentiality, and that no identifying information would be collected. Participants provided electronic informed consent by selecting the agreement option and proceeding to the survey. All responses were collected anonymously and used for research purposes only.

Statistical analysis

Data were entered into Stata 18 (StataCorp, College Station, TX) and analyzed using descriptive and inferential statistics. Chi-square tests were used to compare categorical responses between genders. For multiple-response questions, each response option was coded as selected/not selected and analyzed as a separate binary variable. Spearman’s correlation coefficient was used to examine relationships between ordinal variables. A p value less than 0.05 was considered statistically significant. Because the analyses included multiple comparisons across questionnaire items and demographic variables, the results were interpreted as exploratory. Formal correction for multiple testing was not applied, and statistically significant findings were interpreted alongside their magnitude and clinical or practical relevance.

## Results

Demographic characteristics

A total of 742 participants completed the survey. Of these, 541 (72.9%) were female and 201 (27.1%) were male participants. The age distribution was relatively balanced across different age groups: 123 (16.6%) were 18-24 years, 135 (18.2%) were 25-34 years, 168 (22.6%) were 35-44 years, 164 (22.1%) were 45-54 years, and 152 (20.5%) were 55 years and above. Overall, 475 (64.0%) were married, and 299 (40.3%) held a bachelor's degree.

Most participants, 586 (79.0%), resided in the central region of Saudi Arabia; 352 (47.4%) were in full-time employment, and 244 (32.9%) earned less than 5,000 Saudi Riyal monthly. Chronic diseases affected 210 (28.3%) participants. We observed significant gender differences in marital status (p = 0.008), current job (p < 0.001), and monthly income (p < 0.001). Table 1 presents the demographic characteristics of the participants.

**Table 1:** Demographic data of the survey participants Pearson chi-square tests were used to compare female and male participants across categories for each variable. The test statistic and p value are reported once for each variable SR: Saudi Riyal

Variable	Category	Total sample (n = 742)	Female (n = 541)	Male (n = 201)	Test statistic	p value
Age group	18-24 years	123 (16.58%)	88 (16.27%)	35 (17.41%)	χ² = 6.52	0.163
	25-34 years	135 (18.19%)	106 (19.59%)	29 (14.43%)		
	35-44 years	168 (22.64%)	113 (20.89%)	55 (27.36%)		
	45-54 years	164 (22.10%)	126 (23.29%)	38 (18.91%)		
	55 years and above	152 (20.49%)	108 (19.96%)	44 (21.89%)		
Marital status	Single	223 (30.05%)	167 (30.87%)	56 (27.86%)	χ² = 11.72	0.008
	Married	475 (64.02%)	333 (61.55%)	142 (70.65%)		
	Widow	19 (2.56%)	17 (3.14%)	2 (1.00%)		
	Divorced	25 (3.37%)	24 (4.44%)	1 (0.50%)		
Highest education level	Intermediate or less	16 (2.16%)	14 (2.59%)	2 (1.00%)	χ² = 4.55	0.473
	High school	139 (18.73%)	102 (18.85%)	37 (18.41%)		
	Diploma degree	74 (9.97%)	53 (9.80%)	21 (10.45%)		
	Bachelor's degree	299 (40.30%)	224 (41.40%)	75 (37.31%)		
	Master's or equivalent	112 (15.09%)	80 (14.79%)	32 (15.92%)		
	PhD or equivalent	102 (13.75%)	68 (12.57%)	34 (16.92%)		
Residency region	Central region	586 (78.98%)	427 (78.93%)	159 (79.10%)	χ² = 7.92	0.095
	Eastern region	50 (6.74%)	42 (7.76%)	8 (3.98%)		
	Northern region	13 (1.75%)	8 (1.48%)	5 (2.49%)		
	Southern region	19 (2.56%)	10 (1.85%)	9 (4.48%)		
	Western region	74 (9.97%)	54 (9.98%)	20 (9.95%)		
Current job	Student	139 (18.73%)	103 (19.04%)	36 (17.91%)	χ² = 38.86	<0.001
	Unemployed	194 (26.15%)	171 (31.61%)	23 (11.44%)		
	Employee (part-time)	29 (3.91%)	23 (4.25%)	6 (2.99%)		
	Employee (full-time)	352 (47.44%)	224 (41.40%)	128 (63.68%)		
	Independent	28 (3.77%)	20 (3.70%)	8 (3.98%)		
Monthly income	Less than 5,000 SR	244 (32.88%)	203 (37.52%)	41 (20.40%)	χ² = 41.74	<0.001
	5,000-10,000 SR	126 (16.98%)	102 (18.85%)	24 (11.94%)		
	10,000-15,000 SR	145 (19.54%)	103 (19.04%)	42 (20.90%)		
	15,000-20,000 SR	101 (13.61%)	62 (11.46%)	39 (19.40%)		
	More than 20,000 SR	126 (16.98%)	71 (13.12%)	55 (27.36%)		
Chronic disease	Yes	210 (28.30%)	158 (29.21%)	52 (25.87%)	χ² = 0.80	0.370
	No	532 (71.70%)	383 (70.79%)	149 (74.13%)		

Social media usage patterns

Snapchat was the most frequently used social media platform, reported by 534 (72.0%) participants, followed by Instagram (Meta Platforms), 442 (59.6%), Twitter, 442 (59.6%), and TikTok (ByteDance, Beijing), 335 (45.2%). Significant gender differences were observed in platform preferences. Female participants were more likely than male participants to use Instagram, 374 female participants (69.1%) vs. 68 male participants (33.8%) (p < 0.001), and Snapchat, 420 female participants (77.6%) vs. 114 male participants (56.7%) (p < 0.001), whereas male participants were more likely than female participants to use Twitter, 148 male participants (73.6%) vs. 294 female participants (54.3%) (p < 0.001), and Facebook, 30 male participants (14.9%) vs. 30 female participants (5.5%) (p < 0.001).

Regarding daily time spent on social media, 207 (27.9%) participants reported using it for two to three hours, with no significant gender differences (p = 0.106). A key finding was that the frequency of searching for health-related information (rho = 0.18, p < 0.001) and medication-related information (rho = 0.19, p < 0.001) were positively correlated with time spent on social media.

Most participants reported searching for health-related information on social media sometimes, 329 (44.3%), or frequently, 236 (31.8%), with female participants significantly more likely to do so than male participants (p < 0.001). Similarly, 299 (40.3%) participants sometimes and 164 (22.1%) frequently searched for medication-related information, with significant gender differences (p < 0.001). Table 2 presents the social media usage patterns.

**Table 2:** Social media usage patterns among survey respondents Pearson chi-square tests were used to compare responses by gender. For the multiple-response platform item, each platform was coded as selected/not selected and analyzed as a separate binary item

Question	Response	Total sample (n = 742)	Female (n = 541)	Male (n = 201)	Test statistic	p value
What social media platform do you use?	Facebook	60 (8.09%)	30 (5.55%)	30 (14.93%)	χ² = 17.35	<0.001
	Instagram	442 (59.57%)	374 (69.13%)	68 (33.83%)	χ² = 75.83	<0.001
	Snapchat	534 (71.97%)	420 (77.63%)	114 (56.72%)	χ² = 31.78	<0.001
	TikTok	335 (45.15%)	246 (45.47%)	89 (44.28%)	χ² = 0.08	0.772
	Twitter	442 (59.57%)	294 (54.34%)	148 (73.63%)	χ² = 22.64	<0.001
	Other	147 (19.81%)	108 (19.96%)	39 (19.40%)	χ² = 0.03	0.865
How much time do you spend using social media per day?	Less than one hour	49 (6.60%)	33 (6.10%)	16 (7.96%)	χ² = 7.62	0.106
	1-2 hours	179 (24.12%)	121 (22.37%)	58 (28.86%)		
	2-3 hours	207 (27.90%)	148 (27.36%)	59 (29.35%)		
	3-4 hours	144 (19.41%)	110 (20.33%)	34 (16.92%)		
	More than 4 hours	163 (21.97%)	129 (23.84%)	34 (16.92%)		
Have you used social media to search for health-related information?	Never	57 (7.68%)	35 (6.47%)	22 (10.95%)	χ² = 19.63	<0.001
	Rarely	120 (16.17%)	78 (14.42%)	42 (20.90%)		
	Sometimes	329 (44.34%)	233 (43.07%)	96 (47.76%)		
	Frequently	236 (31.81%)	195 (36.04%)	41 (20.40%)		
Have you used social media to search for medication-related information?	Never	122 (16.44%)	73 (13.49%)	49 (24.38%)	χ² = 17.21	<0.001
	Rarely	157 (21.16%)	113 (20.89%)	44 (21.89%)		
	Sometimes	299 (40.30%)	221 (40.85%)	78 (38.81%)		
	Frequently	164 (22.10%)	134 (24.77%)	30 (14.93%)		
Have you shared or reposted any medication-related information on social media?	Never	313 (42.18%)	224 (41.40%)	89 (44.28%)	χ² = 0.97	0.808
	Rarely	192 (25.88%)	139 (25.69%)	53 (26.37%)		
	Sometimes	184 (24.80%)	139 (25.69%)	45 (22.39%)		
	Frequently	53 (7.14%)	39 (7.21%)	14 (6.97%)		
Have you consulted health communities or online groups for medication-related information?	Never	365 (49.19%)	259 (47.87%)	106 (52.74%)	χ² = 2.48	0.480
	Rarely	144 (19.41%)	104 (19.22%)	40 (19.90%)		
	Sometimes	164 (22.10%)	127 (23.48%)	37 (18.41%)		
	Frequently	69 (9.30%)	51 (9.43%)	18 (8.96%)		

Influence of social media on medication decisions

Regarding regularly used medications, 383 (51.6%) participants reported no regular medication use. The most frequently reported medication categories were other medications, 162 (21.8%); blood pressure and/or lipid-lowering medications, 116 (15.6%); diabetes medications, 93 (12.5%); headache and/or osteoporosis treatment, 90 (12.1%); allergy medications, 76 (10.2%); and sleep medications, 25 (3.4%). Female participants were more likely than male participants to report allergy medications, 64 female participants (11.8%) vs. 12 male participants (6.0%) (p = 0.019); headache and/or osteoporosis treatment, 81 female participants (15.0%) vs. 9 male participants (4.5%) (p < 0.001); and other medications, 129 female participants (23.8%) vs. 33 male participants (16.4%) (p = 0.030), whereas male participants were more likely than female participants to report diabetes medications, 34 male participants (16.9%) vs. 59 female participants (10.9%) (p = 0.028), and blood pressure and/or lipid-lowering medications, 42 male participants (20.9%) vs. 74 female participants (13.7%) (p = 0.016).

When asked about relying on drug information found on social media for drug-purchasing decisions, 209 (28.2%) participants reported sometimes or always doing so, with no significant gender differences (p = 0.348). Regarding the influence of medication-related social media posts on personal decisions about medication use, 299 (40.3%) participants reported sometimes or frequently being influenced, with significant gender differences (p = 0.008). Female participants were more likely than male participants to report being sometimes influenced: 201 female participants (37.2%) vs. 49 male participants (24.4%), whereas male participants were more likely than female participants to report being frequently influenced: 18 male participants (9.0%) vs. 31 female participants (5.7%).

A total of 430 (58.0%) participants preferred visiting a doctor for medication information, while 259 (34.9%) considered both online sources and doctors equally. Regarding concerns about the accuracy of medication information on social media, 614 (82.7%) participants were sometimes or often concerned, with no significant gender differences (p = 0.082) (Table 3).

**Table 3:** Influence of social media on medication decisions Pearson chi-square tests were used to compare responses by gender. For the multiple-response medication item, each medication category was coded as selected/not selected and analyzed as a separate binary item

Question	Response	Total sample (n = 742)	Female (n = 541)	Male (n = 201)	Test statistic	p value
What medications do you use regularly?	Allergy medications	76 (10.24%)	64 (11.83%)	12 (5.97%)	χ² = 5.47	0.019
	Diabetes medications	93 (12.53%)	59 (10.91%)	34 (16.92%)	χ² = 4.83	0.028
	Blood pressure and/or lipid-lowering medications	116 (15.63%)	74 (13.68%)	42 (20.90%)	χ² = 5.79	0.016
	Sleep medications	25 (3.37%)	20 (3.70%)	5 (2.49%)	χ² = 0.66	0.417
	Headache and/or osteoporosis treatment	90 (12.13%)	81 (14.97%)	9 (4.48%)	χ² = 15.14	<0.001
	Other medications	162 (21.83%)	129 (23.84%)	33 (16.42%)	χ² = 4.74	0.030
	No regular medication	383 (51.62%)	269 (49.72%)	114 (56.72%)	χ² = 2.87	0.090
Do you rely on drug information you find on social media when making drug-purchasing decisions?	Never	288 (38.81%)	204 (37.71%)	84 (41.79%)	χ² = 3.30	0.348
	Rarely	245 (33.02%)	187 (34.57%)	58 (28.86%)		
	Sometimes	172 (23.18%)	126 (23.29%)	46 (22.89%)		
	Always	37 (4.99%)	24 (4.44%)	13 (6.47%)		
Do medication-related social media posts influence your personal decisions about medication use?	Never	237 (31.94%)	164 (30.31%)	73 (36.32%)	χ² = 11.73	0.008
	Rarely	206 (27.76%)	145 (26.80%)	61 (30.35%)		
	Sometimes	250 (33.69%)	201 (37.15%)	49 (24.38%)		
	Frequently	49 (6.60%)	31 (5.73%)	18 (8.96%)		
Do recommendations from friends and family about medications on social media influence your decisions about trying those medications?	Never	175 (23.58%)	123 (22.74%)	52 (25.87%)	χ² = 8.78	0.032
	Rarely	252 (33.96%)	176 (32.53%)	76 (37.81%)		
	Sometimes	264 (35.58%)	209 (38.63%)	55 (27.36%)		
	Frequently	51 (6.87%)	33 (6.10%)	18 (8.96%)		
Do you prefer getting information about medications online or visiting your doctor?	Online	40 (5.39%)	27 (4.99%)	13 (6.47%)	χ² = 2.06	0.560
	Visiting the doctor	430 (57.95%)	308 (56.93%)	122 (60.70%)		
	Both are equal	259 (34.91%)	196 (36.23%)	63 (31.34%)		
	Not interested in getting information	13 (1.75%)	10 (1.85%)	3 (1.49%)		
Are you concerned about the accuracy of the information you find on social media about medications?	Never	44 (5.93%)	28 (5.18%)	16 (7.96%)	χ² = 6.70	0.082
	Rarely	84 (11.32%)	55 (10.17%)	29 (14.43%)		
	Sometimes	330 (44.47%)	253 (46.77%)	77 (38.31%)		
	Often	284 (38.27%)	205 (37.89%)	79 (39.30%)		
Do you feel that advertisements promoting medicines on social media are reliable?	Never	235 (31.67%)	170 (31.42%)	65 (32.34%)	χ² = 0.23	0.972
	Rarely	327 (44.07%)	238 (43.99%)	89 (44.28%)		
	Sometimes	163 (21.97%)	121 (22.37%)	42 (20.90%)		
	Always	17 (2.29%)	12 (2.22%)	5 (2.49%)		
Would you prefer to talk to your doctor before making decisions about using medications?	Never	8 (1.08%)	6 (1.11%)	2 (1.00%)	χ² = 0.24	0.971
	Rarely	27 (3.64%)	19 (3.51%)	8 (3.98%)		
	Sometimes	222 (29.92%)	164 (30.31%)	58 (28.86%)		
	Always	485 (65.36%)	352 (65.06%)	133 (66.17%)		

A total of 485 (65.4%) participants reported always preferring to talk to their doctor before making decisions about using medications. Spearman correlation analysis revealed that among female participants, there was a significant positive correlation between age category and perceived reliability of advertisements promoting medicines on social media (rho = 0.122, p = 0.005), and between education level and concern about the accuracy of information found on social media about medications (rho = 0.094, p = 0.028) (Table 4).

**Table 4:** Spearman correlation between age categories, education level, monthly income, and influence of social media on medication decisions Spearman's rho was used to examine correlations between ordinal demographic variables and responses, stratified by gender. Values are reported as rho (p value)

Gender	Item	Age category rho (p value)	Education level rho (p value)	Monthly income rho (p value)
Female	Do you rely on drug information you find on social media when making drug-purchasing decisions?	-0.023 (0.600)	-0.044 (0.306)	-0.041 (0.338)
	Do medication-related social media posts influence your personal decisions about medication use?	-0.060 (0.162)	0.022 (0.610)	-0.026 (0.548)
	Do recommendations from friends and family about medications on social media influence your decisions about trying those medications?	-0.050 (0.250)	-0.001 (0.978)	-0.033 (0.443)
	Are you concerned about the accuracy of the information you find on social media about medications?	-0.030 (0.479)	0.094 (0.028)	0.040 (0.349)
	Do you feel that advertisements promoting medicines on social media are reliable?	0.122 (0.005)	-0.094 (0.028)	-0.014 (0.752)
Male	Do you rely on drug information you find on social media when making drug-purchasing decisions?	-0.049 (0.494)	0.041 (0.560)	-0.060 (0.396)
	Do medication-related social media posts influence your personal decisions about medication use?	-0.090 (0.202)	-0.045 (0.524)	-0.103 (0.144)
	Do recommendations from friends and family about medications on social media influence your decisions about trying those medications?	-0.062 (0.379)	-0.136 (0.055)	-0.102 (0.150)
	Are you concerned about the accuracy of the information you find on social media about medications?	-0.125 (0.078)	0.022 (0.755)	-0.003 (0.966)
	Do you feel that advertisements promoting medicines on social media are reliable?	-0.058 (0.412)	-0.087 (0.222)	-0.137 (0.052)

Behavioral changes, trust in social media, and information-seeking

Regarding behavioral changes based on social media information, the study found that 175 (23.6%) participants reported sometimes, frequently, or always changing their therapy based on social media information, with no significant gender differences (p = 0.131). Similarly, 128 (17.3%) reported sometimes, frequently, or always stopping prescription medications based on social media information, and 133 (17.9%) reported starting medications based on social media information.

Most participants, 393 (53.0%), believed that social media had influenced public attitudes toward medication to some extent, while 198 (26.7%) believed it had done so to a large extent. Nearly half of the participants, 356 (48.0%), felt that social media companies are responsible for regulating drug-related information on their platforms (Table 5).

**Table 5:** Behavioral changes, trust in social media, and information-seeking Pearson chi-square tests were used to compare responses by gender. The test statistic and p value are reported once for each question

Question	Response	Total sample (n = 742)	Female (n = 541)	Male (n = 201)	Test statistic	p value
Have you changed the way you use therapy based on social media information?	Never	363 (48.92%)	260 (48.06%)	103 (51.24%)	χ² = 7.09	0.131
	Rarely	204 (27.49%)	150 (27.73%)	54 (26.87%)		
	Sometimes	127 (17.12%)	101 (18.67%)	26 (12.94%)		
	Frequently	26 (3.50%)	18 (3.33%)	8 (3.98%)		
	Always	22 (2.96%)	12 (2.22%)	10 (4.98%)		
Have you stopped taking prescription medications based on social media information?	Never	428 (57.68%)	306 (56.56%)	122 (60.70%)	χ² = 4.94	0.294
	Rarely	186 (25.07%)	136 (25.14%)	50 (24.88%)		
	Sometimes	88 (11.86%)	72 (13.31%)	16 (7.96%)		
	Frequently	22 (2.96%)	14 (2.59%)	8 (3.98%)		
	Always	18 (2.43%)	13 (2.40%)	5 (2.49%)		
Have you started using medications based on social media information?	Never	435 (58.63%)	312 (57.67%)	123 (61.19%)	χ² = 8.83	0.066
	Rarely	174 (23.45%)	127 (23.48%)	47 (23.38%)		
	Sometimes	101 (13.61%)	82 (15.16%)	19 (9.45%)		
	Frequently	14 (1.89%)	11 (2.03%)	3 (1.49%)		
	Always	18 (2.43%)	9 (1.66%)	9 (4.48%)		
Do you trust drug-related information on social media?	Never	240 (32.35%)	169 (31.24%)	71 (35.32%)	χ² = 15.73	0.003
	Rarely	236 (31.81%)	170 (31.42%)	66 (32.84%)		
	Sometimes	231 (31.13%)	183 (33.83%)	48 (23.88%)		
	Frequently	21 (2.83%)	14 (2.59%)	7 (3.48%)		
	Always	14 (1.89%)	5 (0.92%)	9 (4.48%)		
Do you think social media has influenced public attitudes towards medication?	Not at all	26 (3.50%)	20 (3.70%)	6 (2.99%)	χ² = 5.56	0.135
	To a very limited extent	125 (16.85%)	92 (17.01%)	33 (16.42%)		
	To some extent	393 (52.96%)	297 (54.90%)	96 (47.76%)		
	To a large extent	198 (26.68%)	132 (24.40%)	66 (32.84%)		
Do you feel that social media companies have a responsibility to regulate drug-related information on their platforms?	Yes	356 (47.98%)	255 (47.13%)	101 (50.25%)	χ² = 0.76	0.684
	No	79 (10.65%)	57 (10.54%)	22 (10.95%)		
	I am not sure	307 (41.37%)	229 (42.33%)	78 (38.81%)		
Do you read about new drugs on social media?	Never	147 (19.81%)	103 (19.04%)	44 (21.89%)	χ² = 3.20	0.524
	Rarely	169 (22.78%)	120 (22.18%)	49 (24.38%)		
	Sometimes	274 (36.93%)	210 (38.82%)	64 (31.84%)		
	Frequently	80 (10.78%)	56 (10.35%)	24 (11.94%)		
	Always	72 (9.70%)	52 (9.61%)	20 (9.95%)		
After getting the initial information from social media, have you searched for more information about medications on online platforms?	Never	137 (18.46%)	95 (17.56%)	42 (20.90%)	χ² = 1.21	0.877
	Rarely	130 (17.52%)	95 (17.56%)	35 (17.41%)		
	Sometimes	256 (34.50%)	188 (34.75%)	68 (33.83%)		
	Frequently	115 (15.50%)	85 (15.71%)	30 (14.93%)		
	Always	104 (14.02%)	78 (14.42%)	26 (12.94%)		

A particularly positive finding was that after getting initial information from social media, 475 participants (64.0%) reported sometimes, frequently, or always searching for more information about medications on online platforms. A statistically significant positive correlation was observed between monthly income and searching for more information about medications online after getting initial information from social media among females (rho = 0.100, p = 0.020) (Table 6). However, this association was also weak in magnitude and should be interpreted cautiously.

**Table 6:** Spearman correlation matrix between age categories, education level, monthly income, behavioral changes, trust in social media, and information-seeking Spearman's rho was used to examine correlations between ordinal demographic variables and responses, stratified by gender. Values are reported as rho (p value)

Gender	Item	Age category rho (p value)	Education level rho (p value)	Monthly income rho (p value)
Female	Have you changed the way you use therapy based on social media information?	-0.031 (0.472)	0.056 (0.195)	0.020 (0.638)
	Have you stopped taking prescription medications based on social media information?	0.029 (0.501)	0.045 (0.291)	0.022 (0.614)
	Have you started using medications based on social media information?	-0.014 (0.738)	0.038 (0.376)	0.015 (0.721)
	Do you trust drug-related information on social media?	-0.039 (0.370)	-0.019 (0.665)	0.002 (0.967)
	Do you read about new drugs on social media?	0.053 (0.222)	0.007 (0.864)	0.034 (0.432)
	After getting the initial information from social media, have you searched for more information about medications on online platforms?	0.001 (0.978)	0.082 (0.057)	0.100 (0.020)
Male	Have you changed the way you use therapy based on social media information?	-0.056 (0.428)	0.004 (0.954)	-0.005 (0.939)
	Have you stopped taking prescription medications based on social media information?	0.031 (0.662)	-0.010 (0.883)	0.036 (0.614)
	Have you started using medications based on social media information?	0.021 (0.773)	-0.030 (0.674)	0.013 (0.857)
	Do you trust drug-related information on social media?	-0.112 (0.114)	-0.025 (0.723)	-0.136 (0.053)
	Do you read about new drugs on social media?	0.069 (0.331)	0.046 (0.514)	0.069 (0.331)
	After getting the initial information from social media, have you searched for more information about medications on online platforms?	-0.043 (0.540)	0.099 (0.162)	0.024 (0.735)

## Discussion

This study investigated the role of social media in medication choices among adult users in Saudi Arabia. The findings have significant implications for healthcare practice, policy, and future research.

Social media usage and health information seeking

The findings demonstrate the widespread use of social media platforms among Saudi adults, with Snapchat (Snap Inc., Santa Monica, CA), Instagram, and Twitter being the most popular. The significant gender differences in platform preferences, with female participants favoring Instagram and Snapchat while male participants preferred Twitter and Facebook, highlight the importance of platform-specific approaches when designing health communication strategies.

A key finding of this study is a statistically significant positive association between time spent on social media and the frequency of searches for health- and medication-related information. Although these correlations were statistically significant, their magnitude was weak, and they should therefore be interpreted as modest associations rather than strong predictive relationships. In practical terms, even weak associations may still be relevant at the population level because of the broad reach of social media platforms and users' repeated exposure to health- and medication-related content. This finding is consistent with previous research. Farsi et al. reported that social media can be used to improve the health and knowledge of patients and the public [11]. Furthermore, a systematic review by Cheston et al. demonstrated that social media has an emerging role in promoting medical education [12]. Contrary to the present findings, Saqib and Gazerani reported no association between social media use and over-the-counter medication use among Norwegian university students [13].

The high percentage of participants who reported sometimes (44.34%) or frequently (31.81%) searching for health-related information on social media indicates that these platforms have become significant sources of health information for Saudi adults. This presents both opportunities and challenges for healthcare providers and policymakers.

Several studies have highlighted the complex relationship between social media exposure and medication- or substance-related behaviors among adolescents and young adults. Although social media can provide valuable information and support for individuals struggling with substance use, it can also expose users to pro-drug content and normalize risky behaviors. Systematic analysis of social media content can identify rare adverse events that may not be captured by ordinary regulatory methods [14]. Yang et al. reported that many YouTube videos featuring prescription drug advertisements were not Food and Drug Administration-regulated [15]. Social media may also contribute to several negative consequences related to medication use, including the spread of misinformation and unsafe practices, normalization of drug misuse, ethical concerns involving patient influencers, increased demand and off-label use due to trends, and poorer medication adherence when users rely on social media rather than professional advice [16,17].

Influence on medication decisions

The results suggest that medication-related social media content was associated with self-reported medication decision-making among Saudi adults. While 38.81% of participants reported never relying on drug information found on social media for drug-purchasing decisions, a substantial proportion reported doing so sometimes (23.18%) or always (4.99%). Similarly, 40.29% of participants reported that medication-related social media posts sometimes or frequently influenced their personal decisions about medication use.

The gender differences in self-reported responses to medication-related social media content are particularly noteworthy. Female participants were more likely to report sometimes being influenced by medication-related social media posts (37.15% vs. 24.50%), whereas male participants were more likely to report being frequently influenced (9.00% vs. 5.73%). These findings suggest that gender-specific approaches may be needed when addressing how adults engage with medication-related information on social media.

Despite the influence of social media, it is encouraging that most participants (57.95%) preferred to visit a doctor for medication information, and 65.36% always preferred to talk to their doctor before making decisions about medications. This finding suggests that while social media plays a role in medication decisions, traditional healthcare providers remain the preferred and trusted source of medical advice for most Saudi adults.

Consistent with the present findings, a study among adults in India reported that WhatsApp messages and Facebook posts affected health- and medication-related decisions; 7% of surveyed adults reported starting new medications independently, and 4.2% stopped medicines prescribed by a physician after seeing health-related messages on platforms such as WhatsApp and Facebook [18]. Ertekin et al. reported that 20.9% of adult female acne patients were willing to alter their prescribed treatment based on social media advice, highlighting the potential relevance of social media advice to treatment-related decisions [19].

Recent evidence suggests that the relationship between social media and medication-related behavior remains active, clinically relevant, and not yet fully resolved. A 2026 systematic scoping review of prescription drug promotion by social media influencers identified recurring concerns about misinformation, weak regulatory oversight, inconsistent disclosure practices, and persuasive personal narratives that may blur the boundary between personal experience and paid promotion [20].

In the Saudi context, a recent study on pharmaceutical advertising and public perceptions reported strong public support for stricter regulation and more proactive monitoring of drug advertisements on social media [21]. Previous Saudi studies have also examined the use of social media for medicine-related information, broader online health-information seeking, and the role of social media in choosing healthcare providers [22-24]. Most recently, a 2026 Saudi cross-sectional study examined public use of social networking sites for medicine-related information and their perceived impact on medication-related decisions [25].

The present study complements these recent works by examining medication-related decision-making among adults in Saudi Arabia through a public perspective, linking information-seeking, trust in online medication content, concerns about accuracy, preference for physicians as an information source, and self-reported behavioral changes after exposure to medication-related social media content. This approach helps identify where social media influence may become clinically relevant and where pharmacist- and physician-led counseling can reduce risk.

Trust, concerns, and behavioral changes

Most participants expressed concerns about the accuracy of medication information on social media, with 82.7% reporting that they were sometimes or often concerned. Similarly, most participants felt that advertisements promoting medicines on social media were rarely (44.1%) or never (31.7%) reliable. This healthy skepticism may serve as a protective factor against potentially harmful misinformation.

The positive correlation between education level and concern about the accuracy of medication-related information on social media among female participants (rho = 0.094, p = 0.028) was statistically significant but weak in magnitude. This finding suggests a modest association and should not be interpreted as evidence of a strong predictive relationship. Nevertheless, it may support the importance of health literacy initiatives as part of broader public health strategies.

It is also encouraging that most participants reported never changing their therapy (48.9%), stopping prescription medications (57.7%), or starting medications (58.6%) based on social media information. This finding suggests that while social media may influence attitudes and information-seeking behavior, it may have less impact on medication-taking behavior for most users.

A particularly positive finding was that after getting initial information from social media, 475 participants (64.0%) reported sometimes, frequently, or always searching for more information about medications on online platforms. A significant positive correlation was observed between monthly income and searching for more information about medications online after getting initial information from social media among females (rho = 0.100, p = 0.020) (Table 6).

Although the present study did not directly measure all downstream clinical behaviors, the findings suggest that exposure to medication-related social media content may be associated with some users reporting changes in medication-related decisions or further information-seeking. Similar concerns have been noted in studies of social media advice and treatment decisions [19].

Limitations and future directions

This study has several limitations that should be acknowledged. The cross-sectional design prevents establishing causal relationships between social media use and medication choices; therefore, the findings should be interpreted as associations rather than evidence of causality. The self-reported nature of the data may introduce recall and social desirability biases. The use of social media-based convenience sampling may also have introduced selection bias, as individuals who are more active on social media may have been more likely to participate. In addition, female participants and those from the central region were overrepresented, which may limit the sample's representativeness and the generalizability of the findings to all adults in Saudi Arabia. Multiple statistical comparisons were performed, and formal correction for multiple testing was not applied; therefore, some statistically significant findings may reflect type I error and should be interpreted cautiously. Although the questionnaire demonstrated good overall internal consistency, domain-based reliability coefficients were not calculated separately, which limits interpretation of reliability across specific domains such as trust, influence, behavioral change, and information-seeking. Future studies using longitudinal designs, probability sampling, validated domain-based digital medication-literacy measures, and confirmatory psychometric testing would help clarify causality and strengthen generalizability.

## Conclusions

This study provides important insights into the association between social media use and medication-related decision-making among adult users in Saudi Arabia. The findings indicate that social media is a common source of health and medication information, with a substantial proportion of users reporting that medication-related posts are associated with their medication decisions. However, most participants maintained skepticism about the reliability of such information and preferred consulting healthcare providers for medication advice.

The positive correlations between time spent on social media and health information-seeking behavior suggest that social media may serve as a useful channel for health communication, although these associations were weak in magnitude. The gender differences in platform preferences and self-reported patterns of influence highlight the need for targeted approaches when addressing the role of social media in medication-related decision-making.

These findings have important implications for healthcare providers, policymakers, and social media companies. Healthcare providers should be aware that patients may encounter medication-related information on social media and should be prepared to discuss and clarify information found online. Policymakers should consider strategies to support the accuracy of health information on social media platforms. Social media companies should implement measures to monitor and regulate health-related content on their platforms.

By understanding the relationship between social media use and medication-related decision-making, healthcare providers and public health stakeholders can make better use of social media for health communication while reducing potential risks and supporting safer medication use in the Saudi population.

## Appendices

Appendix A

Arabic Self-Administered Questionnaire

Appendix A includes the Arabic self-administered questionnaire used to collect data from adult social media users in Saudi Arabia.

الجزء الأول: المعلومات العامة

1. ما هو عمرك؟ ○ 18-24 سنة ○ 25-34 سنة ○ 35-44 سنة ○ 45-54 سنة ○ 55 سنة فما فوق

2. ما هو جنسك؟ ○ ذكر ○ أنثى

3. ما هي حالتك الاجتماعية؟ ○ أعزب ○ متزوج ○ مطلق ○ أرمل

4. ما هو أعلى مستوى تعليمي حصلت عليه؟ ○ الشهادة المتوسطة أو أقل ○ الثانوية العامة ○ درجة الدبلوم ○ درجة البكالوريوس ○ ماجستير أو ما يعادله ○ دكتوراه أو ما يعادلها

5. ما هي المنطقة التي تعيش فيها؟ ○ المنطقة الوسطى ○ المنطقة الشمالية ○ المنطقة الشرقية ○ المنطقة الغربية ○ المنطقة الجنوبية

6. ما هو عملك؟ ○ طالب ○ موظف دوام كامل ○ موظف دوام جزئي ○ مستقل ○ بدون عمل

7. ما هو دخلك الشهري؟ ○ أقل من 5000 ريال سعودي ○ 5000-10000 ريال سعودي ○ 10000-15000 ريال سعودي ○ 15000-20000 ريال سعودي ○ أكثر من 20000 ريال سعودي

8. هل لديك أي حالات طبية مزمنة؟ ○ نعم ○ لا

إذا كانت إجابتك نعم، يرجى تحديدها: __________

الجزء الثاني: استخدام وسائل التواصل الاجتماعي

9. ما هي منصات وسائل التواصل الاجتماعي التي تستخدمها؟ يمكنك اختيار أكثر من إجابة. ○ الفيس بوك ○ إنستغرام ○ تويتر ○ سنابشات ○ تيك توك ○ غير ذلك

إذا كانت إجابتك غير ذلك، يرجى تحديدها: __________

10. ما هي مدة استخدامك لوسائل التواصل الاجتماعي الكلية في اليوم؟ ○ أقل من ساعة واحدة ○ ساعة إلى ساعتين ○ ساعتين إلى ثلاث ساعات ○ ثلاث ساعات إلى أربع ساعات ○ أكثر من 4 ساعات

11. هل استخدمت وسائل التواصل الاجتماعي للبحث عن معلومات ذات صلة بالصحة؟ ○ بشكل متكرر ○ أحيانًا ○ نادرًا ○ لا، أبدًا

12. هل استخدمت وسائل التواصل الاجتماعي للبحث عن معلومات ذات صلة بالأدوية؟ ○ بشكل متكرر ○ أحيانًا ○ نادرًا ○ لا، أبدًا

13. هل قمت بمشاركة أو إعادة نشر أي معلومات ذات صلة بالأدوية على وسائل التواصل الاجتماعي؟ ○ بشكل متكرر ○ أحيانًا ○ نادرًا ○ لا، أبدًا

14. هل استشرت مجتمعات الصحة أو المجموعات الإلكترونية الخاصة بالمعلومات ذات الصلة بالأدوية؟ ○ بشكل متكرر ○ أحيانًا ○ نادرًا ○ لا، أبدًا

الجزء الثالث: خيارات الأدوية

15. ما هي الأدوية التي تستخدمها بشكل منتظم؟ يمكنك اختيار أكثر من إجابة. ○ أدوية لعلاج الحساسية ○ أدوية لعلاج السكري ○ أدوية ضغط أو دهون أو كلاهما ○ أدوية لعلاج اضطرابات النوم ○ أدوية لعلاج الصداع أو علاج الهشاشة أو كلاهما ○ أدوية أخرى ○ لا أستخدم أي أدوية بشكل منتظم

16. هل تعتمد على المعلومات المتعلقة بالأدوية التي تجدها على وسائل التواصل الاجتماعي عند اتخاذ قرارات شراء الأدوية؟ ○ نعم، دائمًا ○ نعم، بعض الأحيان ○ لا، نادرًا ○ لا، أبدًا

17. هل تؤثر المشاركات المرتبطة بالأدوية على وسائل التواصل الاجتماعي على قراراتك الشخصية بشأن استخدام الأدوية؟ ○ نعم، كثيرًا ○ نعم، بعض الأحيان ○ لا، نادرًا ○ لا، أبدًا

18. هل تؤثر توصيات الأصدقاء والعائلة حول الأدوية عبر وسائل التواصل الاجتماعي على قراراتك بشأن تجربة تلك الأدوية؟ ○ نعم، كثيرًا وتؤثر بشدة على قراراتي ○ نعم، بعض الأحيان وتؤثر إلى حد ما على قراراتي ○ لا، نادرًا ○ لا، أبدًا

19. هل تفضل الحصول على المعلومات التي تتعلق بالأدوية عبر الإنترنت أو عند زيارة الطبيب؟ ○ عبر الإنترنت ○ عند زيارة الطبيب ○ الاثنين على حد سواء ○ لا أهتم بالحصول على معلومات حول الأدوية

20. هل تشعر بالقلق بشأن صحة المعلومات التي تجدها على وسائل التواصل الاجتماعي المتعلقة بالأدوية؟ ○ نعم، كثيرًا ○ نعم، بعض الأحيان ○ لا، نادرًا ○ لا، أبدًا

21. هل تشعر أن الإعلانات التي تروج للأدوية على وسائل التواصل الاجتماعي موثوقة؟ ○ نعم، دائمًا ○ نعم، بعض الأحيان ○ لا، نادرًا ○ لا، أبدًا

22. هل تفضل التحدث مع الطبيب قبل اتخاذ قرارات بشأن استخدام الأدوية؟ ○ نعم، دائمًا ○ نعم، بعض الأحيان ○ لا، نادرًا ○ لا، أبدًا

23. هل غيرت طريقة استخدامك للعلاج بناءً على معلومات وسائل التواصل الاجتماعي؟ ○ نعم، دائمًا ○ نعم، بشكل متكرر ○ نعم، بعض الأحيان ○ لا، نادرًا ○ لا، أبدًا

24. هل توقفت عن تناول الأدوية الموصوفة بناءً على معلومات وسائل التواصل الاجتماعي؟ ○ نعم، دائمًا ○ نعم، بشكل متكرر ○ نعم، بعض الأحيان ○ لا، نادرًا ○ لا، أبدًا

25. هل بدأت باستخدام الأدوية بناءً على معلومات وسائل التواصل الاجتماعي؟ ○ نعم، دائمًا ○ نعم، بشكل متكرر ○ نعم، بعض الأحيان ○ لا، نادرًا ○ لا، أبدًا

26. هل تثق بالمعلومات ذات الصلة بالأدوية الموجودة على وسائل التواصل الاجتماعي؟ ○ نعم، دائمًا ○ نعم، بشكل متكرر ○ نعم، بعض الأحيان ○ لا، نادرًا ○ لا، أبدًا

27. هل تعتقد أن وسائل التواصل الاجتماعي أثّرت على مواقف الجمهور تجاه الأدوية؟ ○ إلى حد كبير ○ إلى حد ما ○ إلى حد محدود جدًا ○ على الإطلاق

28. هل تشعر أن شركات وسائل التواصل الاجتماعي لها مسؤولية في تنظيم المعلومات ذات الصلة بالأدوية على منصاتها؟ ○ نعم ○ لا ○ لست متأكدًا

29. هل تقرأ عن أدوية جديدة على وسائل التواصل الاجتماعي؟ ○ نعم، دائمًا ○ نعم، بشكل متكرر ○ نعم، بعض الأحيان ○ لا، نادرًا ○ لا، أبدًا

30. هل بحثت عن مزيد من المعلومات حول الأدوية في المنصات الإلكترونية بعد الحصول على المعلومات الأولية من وسائل التواصل الاجتماعي؟ ○ نعم، دائمًا ○ نعم، بشكل متكرر ○ نعم، بعض الأحيان ○ لا، نادرًا ○ لا، أبدًا

Appendix B

English Translation of the Questionnaire

Appendix B provides the English translation of the Arabic self-administered questionnaire. The Arabic version was the version administered to participants.

Part One: General Information

1. What is your age? ○ 18-24 years ○ 25-34 years ○ 35-44 years ○ 45-54 years ○ 55 years or older

2. What is your gender? ○ Male ○ Female

3. What is your marital status? ○ Single ○ Married ○ Divorced ○ Widowed

4. What is the highest educational level you have completed? ○ Intermediate school or less ○ High school ○ Diploma degree ○ Bachelor’s degree ○ Master’s degree or equivalent ○ Doctoral degree or equivalent

5. Which region do you live in? ○ Central region ○ Northern region ○ Eastern region ○ Western region ○ Southern region

6. What is your occupation? ○ Student ○ Full-time employee ○ Part-time employee ○ Self-employed ○ Unemployed

7. What is your monthly income? ○ Less than 5,000 Saudi Riyals ○ 5,000-10,000 Saudi Riyals ○ 10,000-15,000 Saudi Riyals ○ 15,000-20,000 Saudi Riyals ○ More than 20,000 Saudi Riyals

8. Do you have any chronic medical conditions? ○ Yes ○ No

If yes, please specify: __________

Part Two: Social Media Use

9. Which social media platforms do you use? You may select more than one answer. ○ Facebook ○ Instagram ○ Twitter ○ Snapchat ○ TikTok ○ Other

If other, please specify: __________

10. What is your total daily time spent using social media? ○ Less than one hour ○ One to two hours ○ Two to three hours ○ Three to four hours ○ More than four hours

11. Have you used social media to search for health-related information? ○ Frequently ○ Sometimes ○ Rarely ○ No, never

12. Have you used social media to search for medication-related information? ○ Frequently ○ Sometimes ○ Rarely ○ No, never

13. Have you shared or reposted any medication-related information on social media? ○ Frequently ○ Sometimes ○ Rarely ○ No, never

14. Have you consulted health communities or online groups for medication-related information? ○ Frequently ○ Sometimes ○ Rarely ○ No, never

Part Three: Medication Choices

15. Which medications do you use regularly? You may select more than one answer. ○ Medications for allergy treatment ○ Medications for diabetes treatment ○ Blood pressure medications, lipid-lowering medications, or both ○ Medications for sleep disorders ○ Medications for headache treatment, osteoporosis treatment, or both ○ Other medications ○ I do not use any medications regularly

16. Do you rely on medication-related information found on social media when making decisions about purchasing medications? ○ Yes, always ○ Yes, sometimes ○ No, rarely ○ No, never

17. Do medication-related posts on social media influence your personal decisions regarding medication use? ○ Yes, greatly ○ Yes, sometimes ○ No, rarely ○ No, never

18. Do recommendations from friends and family about medications on social media influence your decisions about trying those medications? ○ Yes, greatly, and they strongly influence my decisions ○ Yes, sometimes, and they influence my decisions to some extent ○ No, rarely ○ No, never

19. Do you prefer obtaining medication-related information online or during a doctor visit? ○ Online ○ During a doctor visit ○ Both equally ○ I am not interested in obtaining medication-related information

20. Are you concerned about the accuracy of medication-related information you find on social media? ○ Yes, greatly ○ Yes, sometimes ○ No, rarely ○ No, never

21. Do you feel that advertisements promoting medications on social media are reliable? ○ Yes, always ○ Yes, sometimes ○ No, rarely ○ No, never

22. Do you prefer speaking with a doctor before making decisions about medication use? ○ Yes, always ○ Yes, sometimes ○ No, rarely ○ No, never

23. Have you changed the way you use a treatment based on information from social media? ○ Yes, always ○ Yes, frequently ○ Yes, sometimes ○ No, rarely ○ No, never

24. Have you stopped taking prescribed medications based on information from social media? ○ Yes, always ○ Yes, frequently ○ Yes, sometimes ○ No, rarely ○ No, never

25. Have you started using medications based on information from social media? ○ Yes, always ○ Yes, frequently ○ Yes, sometimes ○ No, rarely ○ No, never

26. Do you trust medication-related information found on social media? ○ Yes, always ○ Yes, frequently ○ Yes, sometimes ○ No, rarely ○ No, never

27. Do you think social media has influenced public attitudes toward medications? ○ To a large extent ○ To some extent ○ To a very limited extent ○ Not at all

28. Do you feel that social media companies have a responsibility to regulate medication-related information on their platforms? ○ Yes ○ No ○ Not sure

29. Do you read about new medications on social media? ○ Yes, always ○ Yes, frequently ○ Yes, sometimes ○ No, rarely ○ No, never

30. Have you searched for additional medication information on online platforms after initially obtaining information from social media? ○ Yes, always ○ Yes, frequently ○ Yes, sometimes ○ No, rarely ○ No, never

## References

[1] (2026). The Global Statistics: Saudi Arabia social media statistics 2025: most popular platforms. https://www.theglobalstatistics.com/saudi-arabia-social-media-users/.

[2] Jia X, Pang Y, Liu LS (2021). Online health information seeking behavior: a systematic review. Healthcare (Basel).

[3] Karim F, Oyewande AA, Abdalla LF, Chaudhry Ehsanullah R, Khan S (2020). Social media use and its connection to mental health: a systematic review. Cureus.

[4] Atkinson DL, Ivanov I (2024). Social media and substance use: how social media shapes and fuels adolescent substance use and how we can respond. J Am Acad Child Adolesc Psychiatry.

[5] Madathil KC, Rivera-Rodriguez AJ, Greenstein JS, Gramopadhye AK (2015). Healthcare information on YouTube: a systematic review. Health Informatics J.

[6] AlSayyari A, AlBuhairan F (2018). Relationship of media exposure to substance use among adolescents in Saudi Arabia: results from a national study. Drug Alcohol Depend.

[7] Alqifari SF, Alrasheedi GA, Alkhiari K (2022). Evaluating social media addiction and self-medicating behavior in adults in Saudi Arabia: a pilot study. Med Sci.

[8] Rowley JE, Johnson FC, Sbaffi L (2017). Gender as an influencer of online health information-seeking and evaluation behavior. J Assoc Inf Sci Technol.

[9] Krosnick JA, Presser S (2010). Question and questionnaire design. Handbook of Survey Research.

[10] Weber W, Gallhofer I, Saris W (2026). Survey Questions, Design Of. SAGE Research Methods Foundations.

[11] Farsi D, Martinez-Menchaca HR, Ahmed M, Farsi N (2022). Social media and health care (part II): narrative review of social media use by patients. J Med Internet Res.

[12] Cheston CC, Flickinger TE, Chisolm MS (2013). Social media use in medical education: a systematic review. Acad Med.

[13] Saqib W, Gazerani P (2024). Social media use and consumption of prescription-free medications for anxiety, sleep, and pain among Norwegian university students. Eur J Investig Health Psychol Educ.

[14] Gijsen V, Maddux M, Lavertu A (2020). #Science: the potential and the challenges of utilizing social media and other electronic communication platforms in health care. Clin Transl Sci.

[15] Yang M, Seo J, Patel A, Sansgiry SS (2012). Content analysis of the videos featuring prescription drug advertisements in social media: YouTube. Drug Inf J.

[16] George DR, Rovniak LS, Kraschnewski JL (2013). Dangers and opportunities for social media in medicine. Clin Obstet Gynecol.

[17] Giustini D, Ali SM, Fraser M, Kamel Boulos MN (2018). Effective uses of social media in public health and medicine: a systematic review of systematic reviews. Online J Public Health Inform.

[18] Rehman T, Mallick A, Ghosh T, Ahamed F (2024). Influence of social media on health-related decision-making among adults attending an outpatient department of a tertiary care centre in India: a cross-sectional analytical study. Natl Med J India.

[19] Ertekin SS, Salici NS, Manav Bas V (2024). Influence of social media and internet on treatment decisions in adult female acne patients: a cross-sectional survey study. Dermatol Pract Concept.

[20] Gell S, Dave S, Willis E, Vitale EJ, Woloshin S, Heiss R (2026). Prescription drug promotion by social media influencers: a systematic scoping review. JAMA Netw Open.

[21] Alnuhait MA, Althobaiti HA, Alharbi MH (2024). Pharmaceutical advertising and public perceptions in Saudi Arabia. Pharmacy (Basel).

[22] Alhaddad MS (2018). The use of social media among Saudi residents for medicines related information. Saudi Pharm J.

[23] AlMuammar SA, Noorsaeed AS, Alafif RA, Kamal YF, Daghistani GM (2021). The use of internet and social media for health information and its consequences among the population in Saudi Arabia. Cureus.

[24] Hariri NH, Alruwais AT, Sodagar WM (2025). Association between social media use and patients' choice of medical practitioners among the general population in Saudi Arabia: a cross-sectional study. Healthcare (Basel).

[25] Alhomoud F, Alhomoud FK, Al Muslim A (2026). Public views, patterns, and impacts of social networking site use for medicine-related information in Saudi Arabia: a cross-sectional study. J Public Health Res.

